# Passive Smoking Is Associated with the Risk of Functional Bowel Disorders Among College Freshmen

**DOI:** 10.3390/healthcare12232477

**Published:** 2024-12-07

**Authors:** Jinlu Guo, Fan Du, Chaofan Duan, Can Chen, Jingze Yang, Xin Yang, Shi Liu, Tao Bai, Xiaohua Hou

**Affiliations:** Department of Gastroenterology, Union Hospital, Tongji Medical College, Huazhong University of Science and Technology, 1277 Jiefang Avenue, Wuhan 430022, China; d202181839@hust.edu.cn (J.G.);

**Keywords:** functional bowel disorders, risk factors, passive smoking, college freshmen

## Abstract

**Background**: Functional bowel disorders (FBDs) have detrimental effects on young adults, but the risk factors were not fully explored. This study aimed to investigate the prevalence and potential risk factors of FBDs in college freshmen, including, in particular, the association between passive smoking and the risk and symptoms of FBDs. **Methods**: A cross-sectional study was conducted in September 2019 in freshmen of Huazhong University of Science and Technology with a random cluster sampling method. Validated questionnaires were voluntarily completed by participants. Rome IV criteria were applied for the diagnosis of FBDs. Univariate analysis and multivariate logistic regression analysis (Model 1: unadjusted; Model 2: adjusted for age and sex; Model 3: adjusted for age, sex, intake frequency of coffee and juice, regular exercise, total sedentary time, sleep quality, interpersonal relationship, and SLSI scores) were performed to determine the potential risk factors of FBDs. **Results**: A total of 3074 participants were included in this study, among whom 236 college freshmen were diagnosed with FBDs. There was a positive relationship between passive smoking and the risk of FBDs (crude odds ratio [OR] = 2.084, 95% confidence interval [CI]: 1.480, 2.936, Model 1; adjusted OR = 1.825, 95%CI: 1.245, 2.675, Model 3). Moreover, the symptoms of hard stool, exertion, and sensation of obstruction in defecation were more frequent in passive smokers than non-passive smokers among FBD patients. Meanwhile, diarrhea was comparable between passive smokers and non-passive smokers among FBD patients. **Conclusions**: In the present study, around 7.68% of college freshmen were found to have FBDs. Passive smoking was positively associated with the risk of FBDs. Furthermore, passive smoking was significantly associated with constipation-related symptoms rather than diarrhea among FBD patients.

## 1. Introduction

Functional bowel disorders (FBDs) are chronic conditions stemming from the dysregulation of the gut–brain axis and characterized by persistent and recurring gastrointestinal symptoms in the lower alimentary tract. Five categories are recognized according to Rome IV: irritable bowel syndrome (IBS), functional diarrhea (FDr), functional constipation (FC), functional abdominal bloating/distention (FAB/D), and unspecific FBD [1]. They are prevalent and challenging digestive disorders worldwide. The pooled prevalence rate of FBDs was estimated to be around 33.4% in largescale population research among 33 countries [2]. In China, previous studies reported a prevalence of around 20% in the general population [2,3,4]. Nowadays, studies have found the detrimental effects of FBDs on young people, including negative self-esteem, emotional problems, and helplessness in their lives and studies [5,6]. But, the risk factors of FBDs in young people are not fully understood. Therefore, it is necessary to investigate the current status of FBDs and explore the potential risk factors among young populations. As a part of young populations, college freshmen are at a transitional stage from adolescence to adulthood, experiencing uncertainties in both physical and psychological development [7,8,9,10]. Thus, compared to the general adult population, college freshmen are more susceptible to the influence of environmental factors [8]. Identifying the relevant risk factors may contribute to avoiding them, thus reducing the risk of FBDs and improving the symptoms of FBDs.

Among the environmental risk factors, passive smoking is worthy of attention. To date, passive smoking suffered from parent active smokers is more common than active smoking among young adults in China [11]. Some studies have suggested that active smoking is associated with functional gastrointestinal disorders [12,13], while there was no study focusing on passive smoking although it is a common risk factor in multisystem diseases. Substantial evidence demonstrated that passive smoking was associated with oxidative damage, impairment of the intestinal barrier and immune function, and imbalance of microbiota [14]. These injury mechanisms were also involved in the onset of symptoms relating to FBDs [15]. However, whether passive smoking was associated with FBDs in college freshmen was unknown.

Thus, this study aimed to investigate the association between passive smoking and FBDs in young college freshmen. Furthermore, we aimed to explore the association between passive smoking and FBD symptoms.

## 2. Materials and Methods

### 2.1. Participants

A cross-sectional study was conducted in September 2019 in freshmen of Huazhong University of Science and Technology (HUST). A random cluster sampling method was adopted. After freshmen registration, classes at different campuses or departments were selected randomly. Informed consent forms were obtained from all participants. Questionnaires were filled out by all participants voluntarily and anonymously. Finally, freshmen of HUST with or without FBDs who filled out the questionnaires were included. Participants with symptom(s) attributable to their medical history, concurrent functional gastric disorders, or whose data were insufficient for a diagnosis of FBD, and active smokers, ex-smokers, and subjects with unclear smoking history were excluded. Considering that the prevalence of FBDs varied from 13.2% to 18.3% in relatively young adults [2,16], according to the established formula, the sample size of 3074 included in the study is sufficient to achieve a precision of ±2.5% with a 95% confidence interval. The study was approved by the Ethics Committee of Tongji Medical College, Huazhong University of Science and Technology (No: IORG0003571) and was in accordance with the Declaration of Helsinki.

### 2.2. Questionnaires

Four parts focused on demographic information, general health status, digestive symptoms, and student-life stress inventory (SLSI) were included.

1. Demographic information included sex, age, height, and weight.
2. General health status contained medical history (previous diseases, medication, and allergy history) and living habits (smoking status, alcohol consumption, dietary habits, exercise, total sedentary time, sleep quality, and interpersonal relationships). Passive smoking was defined as inhaling smoke produced by others or the side-stream of conventional cigarettes for more than 15 min per day at least once a week [17]. Passive smoking exposure was meticulously recorded in terms of minutes per day, days per week, years of exposure, and main exposed places. Active smoking was defined as actively smoking at least one conventional cigarette per day on average for at least half a year and currently smoking; ex-smoking was defined as actively smoking at least one conventional cigarette per day on average for at least half a year and quitting smoking; never smoking was considered as a healthy level, which was defined as smoking less than one cigarette per day on average for less than six months or no smoking behavior. Alcohol consumption was defined as drinking at least once per day for more than six months. In the dietary habits inventory, the frequency of intake for each food or beverage was divided into “less than once a month”, “once a month to four times a week” and “no less than four times a week” groups to avoid extreme cases. Regular exercise was described as exercising more than 20 min per day and at least thrice per week over the past six months. Total sedentary duration in the past month was investigated and divided into “less than 42 h per week” and “no less than 42 h per week” groups according to the 50th percentile. Sleep quality and interpersonal relationships were both divided into three grades: good, general, and poor.
3. A digestive symptom inventory was based on Rome IV criteria [15]. Simultaneously, we recorded the frequency of each symptom of FBDs (abdominal pain, hard stool, decreased defecation, exertion in defecation, sensation of incomplete emptying in defecation, sensation of obstruction in defecation, aid of manipulation, abdominal bloating/distention and diarrhea) over the last three months. Then, we divided participants into “Yes” and “No” groups in terms of whether they had the symptoms, and we divided FBD patients into “high frequency” and “low frequency” groups according to the 50th percentile.
4. The SLSI consisted of 51 items for evaluating the stress of students, and higher scores indicated a higher level of stress [18].

### 2.3. Statistical Analysis

A Kolmogorov–Smirnov test was performed to assess whether the continuous variables were normally distributed or not. The normally distributed data were presented as mean ± standard deviation (SD) and non-normally distributed data were presented as medians (interquartile ranges), and categorical variables were presented as numbers and percentages. The variables were tested using the Kruskal–Wallis test, the χ^2^ test with or without correction for continuity, or Fisher’s exact test, as appropriate. Age, sex, and variables whose *p* values were less than 0.1 in univariate analysis were included in the following logistic regression models. (1). Unadjusted. (2). Adjusted for age and sex. (3). Adjusted for age, sex, intake of coffee and juice, regular exercise, total sedentary duration, sleep quality, interpersonal relationships, and SLSI scores. A two-tailed *p* value of less than 0.05 was considered to be of statistical significance. Statistical analyses were performed using SPSS version 23.0 (IBM, Armonk, NY, USA).

## 3. Results

### 3.1. The Prevalence of FBDs and General Characteristics of Participants

**Figure 1:**
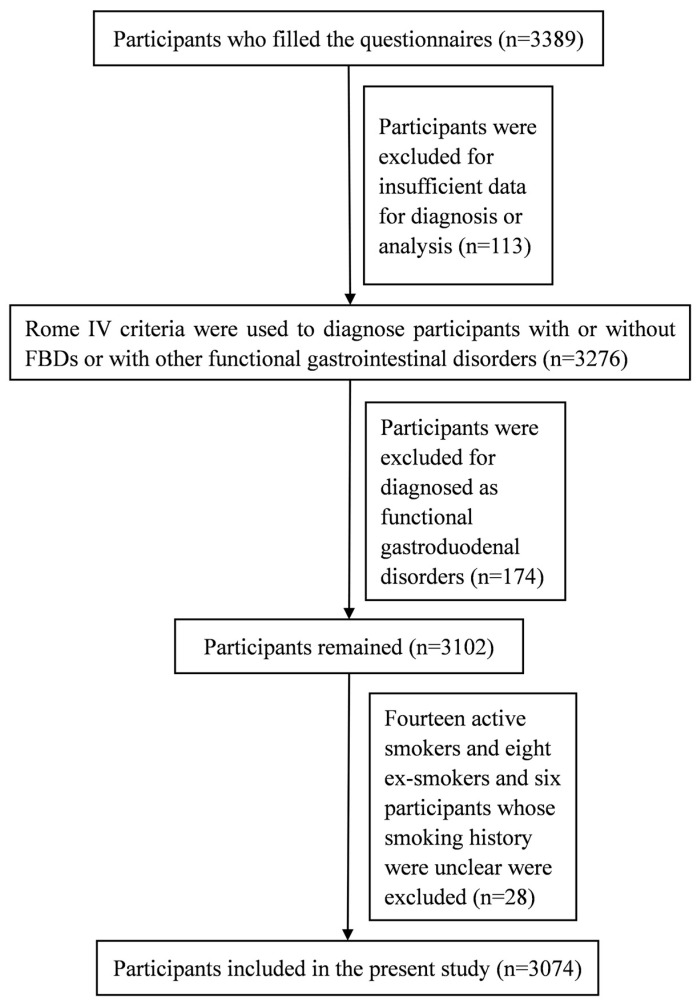
The flow diagram of participants’ inclusion.

**Figure 2:**
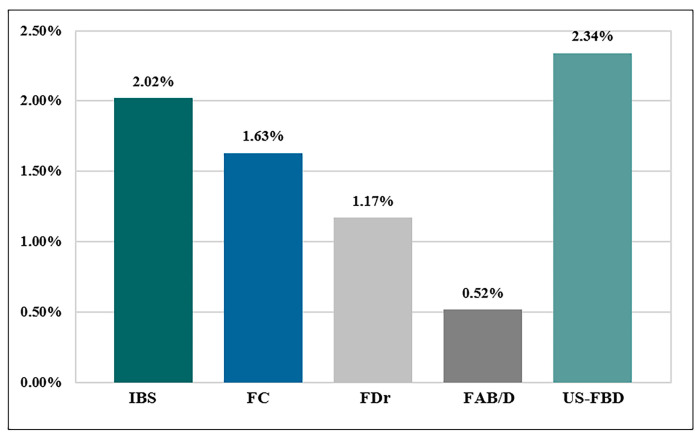
The prevalence of each subtype of FBD in college freshmen. IBS: irritable bowel syndrome. FC: functional constipation. FDr: functional diarrhea. FAB/D: functional abdominal bloating/distention. US-FBD: unspecific functional bowel disorders.

A total of 3389 participants filled out the questionnaires, and 113 were excluded for insufficient data for diagnosis and analysis. Participants with gastroduodenal disorders (174), active smokers (14), ex-smokers (8), and subjects with unclear smoking history (6) were excluded. Finally, 3074 participants were included (shown in Figure 1). The median age of the participants was 18.3 years (shown in Table 1), ranging from 16 to 22. Among these college freshmen, 236 (7.68% of 3074) participants were diagnosed with FBDs, including irritable bowel syndrome (2.02%), functional diarrhea (1.17%), functional constipation (1.63%), functional abdominal bloating/distention (0.52%), and unspecific FBD (2.34%) (shown in Figure 2).

### 3.2. Univariate Analysis of College Freshmen with and Without FBDs

As shown in Table 1, female sex, intake of coffee at moderate frequency, long sedentary time (>42 h a week), poor sleep quality, and poor interpersonal relationships, as well as passive smoking, were positively associated with FBDs. Regular exercise was negatively associated with FBDs. SLSI scores were much higher in FBD patients compared to non-FBD patients, which indicated that a higher stress level was positively associated with FBDs.

### 3.3. Multivariate Logistic Regression Analysis of the Association Between Passive Smoking and FBDs

To clarify the association between passive smoking and the risk of FBDs, multivariate logistic regression analysis was performed to exclude the influence of possible confounding factors (variables whose *p* values were less than 0.1 in univariate analysis). In Table 2, the crude OR for passive smokers vs. non-passive smokers was 2.084 (Model 1; 95%CI: 1.480, 2.936). After adjusted for sex and age, the OR was 1.952 (Model 2; 95%CI: 1.371, 2.780). The association between passive smoking and FBDs was still sustained with the OR of 1.825 (Model 3; 95%CI: 1.245, 2.675) after adjusting for age, sex, intake of coffee and juice, regular exercise, total sedentary duration, sleep quality, interpersonal relationships, and SLSI scores.

Other factors, namely female sex, intake of coffee at moderate frequency, poor sleep quality, poor interpersonal relationships, long sedentary time (>42 h per week), and higher SLSI scores were still associated with FBDs in multivariate logistic regression analysis models (shown in Table 2).

Furthermore, the differences in minutes per day, days per week, and years of exposure to passive smoking between passive smokers and non-passive smokers were of statistical significance. There was no statistical difference among places of exposure to passive smoking in subjects with or without FBDs (shown in Table 3). To clarify whether there was an accumulative effect between passive smoking and FBDs, multivariate logistic regression analysis was also used to exclude the influence of confounding factors (models were the same and mentioned in the Method section), and the results (shown in Table 3) indicated that compared to those exposed for than half an hour per day, young adults being exposed to passive smoking for more than half an hour per day was not associated with higher risk of FBDs (adjusted OR = 1.394, 95%CI: 0.403, 4.820). However, exposure to passive smoking for more than 3 days a week (adjusted OR = 2.180, 95%CI: 1.203, 3.949) was positively associated with FBDs compared to no more than 2 days a week (adjusted OR = 1.589, 95%CI: 0.997, 2.534). Similarly, subjects with passive smoking for over 10 years also showed a positive association with FBDs (adjusted OR = 1.676, 95%CI: 1.022, 2.749), compared to those whose exposure to passive smoking was for no more than 10 years (adjusted OR = 1.973, 95%CI: 1.118, 3.483).

### 3.4. The Association Between Passive Smoking and Symptoms of FBDs

Compared to non-passive smokers, passive smokers were more likely to be associated with digestive symptoms of abdominal bloating/distention, hard stool, decreased defecation frequency, exertion, and sensation of obstruction in defecation (shown in Table 4).

Furthermore, FBD patients suffering from hard stool, exertion, and sensation of obstruction in defecation at higher frequency were more common in passive smokers than in non-passive smokers. But, passive smoking was not associated with the frequency of abdominal bloating/distention or decreased defecation among FBD patients (shown in Table 5). Whether in the general population or in FBD patients, passive smoking was not associated with abdominal pain, diarrhea, sensation of incomplete emptying in defecation, or aid of manipulation (shown in Table 4 and Table 5).

## 4. Discussion

In the present study, we demonstrated that passive smoking was independently associated with FBDs in college freshmen after adjusting for age, sex, coffee and juice consumption, regular exercise, total sedentary duration, sleep quality, interpersonal relationships, and stress level. A significant accumulative effect was also observed between total passive smoking time and the risk of FBDs. Additionally, we found that passive smoking was associated with constipation-related symptoms rather than diarrhea.

To the best of our knowledge, the present study is the first to explore the association between passive smoking and FBDs. And, we demonstrated that passive smoking was independently associated with FBDs. Previous studies mainly focused on the association between active smoking and gastrointestinal diseases and revealed that active smoking was associated with functional gastrointestinal disorders [12,13], while there was no study exploring the association between passive smoking and FBDs. However, quite a few studies suggested that passive smoking was associated with non-functional digestive diseases, such as ulcerative colitis, Crohn’s disease, and gastrointestinal cancers [19,20]. The present study verified the association between passive smoking and FBDs and provided a clue for further study.

A significant accumulative effect between passive smoking and FBDs was observed in the present study. Recently, a study suggested a dose–response effect of passive smoking on the risk of ulcerative colitis [14]. The accumulative effect of passive smoking also has been demonstrated in other organic diseases, such as chronic obstructive pulmonary disease, congenital heart disease, and neoplastic diseases [21,22,23]. However, the places of exposure to passive smoking were not associated with FBDs in this study, which was inconsistent with the result reported by Atsushi Nishikawa [14]. She investigated the relationship between the places of exposure to passive smoking and ulcerative colitis in the general adult population and verified that people exposed to passive smoking at home were more likely to have ulcerative colitis than those in workplaces. This indicated that passive smoking may act differently in different populations or gastrointestinal diseases. In this study, passive smoking has been revealed to be independently associated with FBDs with a cumulative effect, regardless of the places of exposure to passive smoking.

Passive smokers were more likely to have constipation-related symptoms. And, FBD patients with exposure to passive smoking tended to suffer a higher frequency of constipation-related symptoms, including hard stool, exertion, and sensation of obstruction in defecation compared to non-passive smokers. But, passive smoking was not associated with diarrhea. However, in another study [24], the result suggested that there may be an association between active smoking and diarrhea but not constipation. The reason why the results showed the opposite is unclear and worthy of further study.

We also demonstrated that female sex, intake of coffee at moderate frequency, poor sleep quality, poor interpersonal relationships, long sedentary time, and higher stress levels were associated with FBDs among college freshmen. This is consistent with many previously published studies. Epidemiological data revealed that FBDs were more prevalent in females compared to males [2,15]. Poor sleep quality, poor interpersonal relationships, stress, and a sedentary lifestyle may contribute to FBDs through detrimental effects on autonomic nervous function, the brain–gut axis, holistic metabolic conditions, and gut microbiota diversity [25,26,27,28,29]. Little was known about the roles of coffee consumption in FBDs.

In conclusion, passive smoking was independently associated with the risk of FBDs with an accumulative effect. Among FBD patients, passive smoking was significantly associated with constipation-related symptoms rather than diarrhea. Thus, the study provides a clue for future investigations to verify whether there is a causal relationship between passive smoking and the risk of FBDs.

There are several limitations to the present study. First of all, it was a cross-sectional study, the causal relationship is not certain, and recall bias could not be avoided. Secondly, the study was only carried out in one university. But, the results did provide a clue regarding the association between passive smoking and FBDs. Thirdly, due to the small sample size of active smokers, we did not investigate the association between active smoking and FBDs and only drew conclusions based on a population of selective never-smokers among young college freshmen. Thus, more comprehensive evidence is needed to verify the relationship.

## Figures and Tables

**Table 1:** Characteristics and univariate analysis of college freshman with FBDs (N = 3074).

Variables		Total (%)	FBD (%)	Non-FBD (%)	χ^2^	*p* Value
Sex ^†^	Male	2382 (78.4)	167 (71.4)	2215 (78.9)	7.299	0.007
	Female	658 (21.6)	67 (28.6)	591 (21.1)		
Age (median [IQR]) *	-	18.3 [17.9–18.7]	18.3 [18.1–18.7]	18.3 [19.7–18.7]		0.351
Allergy ^†^	Yes	778 (25.3)	69 (29.2)	709 (25.0)	2.087	0.149
	No	2296 (74.7)	167 (70.8)	2129 (75.0)		
Alcohol ^§^	Yes	49 (1.6)	4 (1.7)	45 (1.6)	-	1.000
	No	3018 (98.4)	232 (98.3)	2786 (98.4)		
Diet						
Frumentum ^§^	<1/mo	4 (0.1)	0 (0.0)	4 (0.1)	-	0.245
	1/mo–3/wk	130 (4.2)	5 (2.1)	125 (4.4)		
	>3/wk	2937 (95.6)	231 (97.9)	2706 (95.4)		
Coarse grains ^†^	<1/mo	272 (8.9)	22 (9.4)	250 (8.8)	3.747	0.154
	1/mo–3/wk	2112 (69.0)	172 (73.5)	1940 (68.6)		
	>3/wk	678 (22.1)	40 (17.1)	638 (22.6)		
Fresh vegetables ^†^	<1/mo	29 (0.9)	0 (0)	29 (1.0)	3.078	0.215
	1/mo–3/wk	1064 (34.7)	77 (32.6)	987 (34.8)		
	>3/wk	1976 (64.4)	159 (67.4)	1817 (64.1)		
Farmed meat ^†^	<1/mo	13 (0.4)	3 (1.3)	10 (0.4)	4.405	0.111
	1/mo–3/wk	302 (9.8)	22 (9.3)	280 (9.9)		
	>3/wk	2752 (89.7)	211 (89.4)	2541 (89.8)		
Aquatic products ^†^	<1/mo	330 (10.8)	24 (10.2)	306 (10.8)	0.245	0.885
	1/mo–3/wk	2259 (73.7)	172 (73.2)	2087 (73.7)		
	>3/wk	478 (15.6)	39 (16.6)	439 (15.5)		
Eggs ^†^	<1/mo	23 (0.7)	2 (0.9)	21 (0.7)	1.348	0.510
	1/mo–3/wk	580 (18.9)	51 (21.7)	529 (18.7)		
	>3/wk	2464 (80.3)	182 (77.4)	2282 (80.6)		
Smoked products ^†^	<1/mo	924 (30.1)	70 (29.7)	854 (30.2)	1.535	0.464
	1/mo–3/wk	1940 (63.3)	146 (61.9)	1794 (63.4)		
	>3/wk	201 (6.6)	20 (8.5)	181 (6.4)		
Drink						
Water ^†^	<1/mo	56 (1.8)	8 (3.4)	48 (1.7)	4.296	0.117
	1/mo–3/wk	124 (4.0)	12 (5.1)	112 (4.0)		
	>3/wk	2887 (94.1)	216 (91.5)	2671 (94.3)		
Green tea ^†^	<1/mo	1747 (58.0)	130 (55.8)	1617 (58.2)	0.544	0.762
	1/mo–3/wk	1085 (36.0)	89 (38.2)	996 (35.8)		
	>3/wk	180 (6.0)	14 (6.0)	166 (6.0)		
Black tea ^†^	<1/mo	1625 (53.8)	111 (47.6)	1514 (54.3)	3.890	0.143
	1/mo–3/wk	1238 (41.0)	109 (46.8)	1129 (40.5)		
	>3/wk	160 (5.3)	13 (5.6)	147 (5.3)		
Cola ^†^	<1/mo	759 (24.9)	49 (20.9)	710 (25.2)	2.844	0.241
	1/mo–3/wk	1812 (59.4)	143 (60.9)	1669 (59.3)		
	>3/wk	479 (15.7)	43 (18.3)	436 (15.5)		
Coffee ^†^	<1/mo	2042 (67.5)	135 (57.9)	1907 (68.3)	10.875	0.004
	1/mo–3/wk	845 (27.9)	86 (36.9)	759 (27.2)		0.001 (v.s. < 1/mo)
	>3/wk	139 (4.6)	12 (5.2)	127 (4.5)		0.359 (v.s. < 1/mo)
Juice ^†^	<1/mo	547 (18.0)	30 (12.8)	517 (18.4)	5.829	0.054
	1/mo–3/wk	1884 (62.0)	148 (63.2)	1736 (61.9)		
	>3/wk	607 (20.0)	56 (23.9)	551 (19.7)		
Exercise ^†^	Little	1318 (43.0)	123 (52.1)	1195 (42.2)	8.698	0.003
	Regular	1748 (57.0)	113 (47.9)	1635 (57.8)		
Sedentary duration ^†^	<42 h/w	1386 (49.6)	83 (37.9)	1303 (50.6)	13.027	<0.001
	≥42 h/w	1408 (50.4)	136 (62.1)	1272 (49.4)		
Sleep quality ^†^	Good	1628 (53.4)	95 (40.6)	1533 (54.5)	36.317	<0.001
	General	1321 (43.3)	118 (50.4)	1203 (42.8)		
	Bad	99 (3.2)	21 (9.0)	78 (2.8)		
Relationships ^†^	Hardly	214 (7.0)	40 (17.1)	174 (6.1)	51.127	<0.001
	General	686 (22.4)	67 (28.6)	619 (21.9)		
	Always	2165 (70.6)	127 (54.3)	2038 (72.0)		
SLSI (median [IQR]) *	-	61.5 [46.0–83.0]	74.7 [60.3–90.3]	60.0 [45.0–82.0]		<0.001
Passive smoking ^†^	Yes	345 (11.8)	47 (20.5)	298 (11.0)	18.349	<0.001
	No	2587 (88.2)	182 (79.5)	2405 (89.0)		

FBDs: functional bowel diseases; IQR: interquartile ranges. ^†^: Pearson’s χ^2^ test; *: Kruskal–Wallis test; ^§^: Fisher’s exact test. *p* < 0.05 was considered to be of statistical significance.

**Table 2:** Multiple logistic regression analyses of the relationship between FBDs and passive smoking and other confounding risk factors (N = 3074).

Variables	Crude OR (95%CI, Model 1)	Adjusted OR (95%CI, Model 2)	Adjusted OR (95%CI, Model 3)	*p* Value (Referred to Model 3)	
Passive smoking					
Never	1.00	1.00	1.00	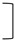	
Yes	2.084 (1.480, 2.936)	1.952 (1.371, 2.780)	1.825 (1.245, 2.675)		0.002
Gender					
Male	1.00	1.00	1.00	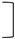	
Female	1.504 (1.117, 2.025)	1.506 (1.107, 2.049)	1.421 (1.015, 1.990)		0.041
Coffee					
<1/mo	1.00	1.00	1.00	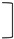	
1/mo–3/wk	1.601 (1.206, 2.125)	-	1.489 (1.081, 2.051)		0.015
>3/wk	1.335 (0.720, 2.474)	-	0.994 (0.503, 1.966)		
Juice					
<1/mo	1.00	1.00	1.00	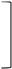	
1/mo–3/wk	1.469 (0.980, 2.202)	-	1.586 (1.006, 2.502)		
>3/wk	1.751 (1.106, 2.773)	-	1.851 (1.104, 3.102)		0.020
Sleep quality					
Good	1.00	1.00	1.00	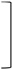	
General	1.583 (1.196, 2.095)	-	1.223 (0.895, 1.671)		
Poor	4.345 (2.571, 7.341)	-	2.137 (1.105, 4.131)		0.024
Interpersonal relationships					
Good	1.00	1.00	1.00	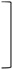	
General	1.737 (1.275, 2.367)	-	1.437 (1.013, 2.040)		
Poor	3.689 (2.504, 5.436)	-	2.213 (1.385, 3.535)		<0.001
Sedentary time					
<42 h/w	1.00	1.00	1.00	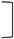	
≥42 h/w	1.678 (1.264, 2.229)	-	1.407 (1.036, 1.911)		0.029
SLSI	1.014 (1.009, 1.018)	-	1.009 (1.004, 1.014)		0.001

FBDs: functional bowel disorders. OR: odds ratio. CI: confidence intervals. Model 1: unadjusted. Model 2: adjusted for age and gender. Model 3: Adjusted for age, gender, coffee, juice, exercise, total sedentary duration, sleep quality, interpersonal relationships, and SLSI scores. *p* < 0.05 was considered to be of statistical significance.

**Table 3:** Comparison of passive smoking characteristics between groups with and without FBDs (N = 3074).

Passive Smoking Characteristics	Total (%)	FBD (%)	Non-FBD (%)	*p* Value	Crude OR (95%CI, Model 1)	Adjusted OR (95%CI, Model 2)	Adjusted OR (95%CI, Model 3)		*p* Value (Referred to Model 3)
Daily time ^†^				<0.001					
Never	2587 (88.5)	182 (80.2)	2405 (89.2)		1.00	1.00	1.00	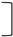	
≤30 min	302 (10.3)	41 (18.1)	261 (9.7)		2.076 (1.445, 2.982)	1.923 (1.321, 2.798)	1.799 (1.202, 2.693)		0.004
>30 min	33 (1.1)	4 (1.8)	29 (1.1)		1.823 (0.634, 5.241)	1.874 (0.648, 5.416)	1.394 (0.403, 4.820)		
Days per week ^†^				<0.001					
Never	2586 (88.3)	182 (79.8)	2404 (89.0)		1.00	1.00	1.00	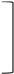	
≤2 days	238 (8.1)	29 (12.7)	209 (7.7)		1.833 (1.208, 2.780)	1.665 (1.075, 2.578)	1.589 (0.997, 2.534)		
>3 days	105 (3.6)	17 (7.5)	88 (3.3)		2.552 (1.486, 4.382)	2.478 (1.438, 4.270)	2.180 (1.203, 3.949)		0.010
Years ^†^				<0.001					
Never	2587 (89.0)	182 (80.5)	2405 (89.7)		1.00	1.00	1.00	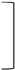	
≤10 years	199 (6.8)	25 (11.1)	174 (6.5)		1.899 (1.216, 2.964)	1.773 (1.116, 2.816)	1.676 (1.022, 2.749)		
>10 years	122 (4.2)	19 (8.4)	103 (3.8)		2.438 (1.461, 4.068)	2.230 (1.315, 3.780)	1.973 (1.118, 3.483)		0.019
Places of exposure ^†^				0.496	-	-	-		
Office	59 (13.3)	6 (9.4)	53 (14.0)		-	-	-		
Home	239 (54.0)	34 (53.1)	205 (54.1)		-	-	-		
Others	145 (32.7)	24 (37.5)	121 (31.9)		-	-	-		

FBDs: functional bowel disorders. OR: odds ratio. CI: confidence intervals. ^†^: Pearson’s χ^2^ test; *p* < 0.05 was considered to be of significance.

**Table 4:** The association between passive smoking and FDB symptoms (N = 3074).

Symptoms	Passive Smoking (%)		χ^2^	*p* Value
Abdominal pain ^†^	Yes	Never		
Yes	147 (42.7)	972 (37.8)	3.151	0.076
No	197 (57.3)	1601 (62.2)		
Abdominal bloating and/or distention ^†^	Yes	Never		
Yes	111 (32.5)	639 (25.0)	8.869	0.003
No	231 (67.5)	1922 (75.0)		
Hard stool ^†^	Yes	Never		
Yes	226 (65.5)	1486 (57.8)	7.499	0.006
No	119 (34.5)	1086 (42.2)		
Decreased defecation ^†^	Yes	Never		
Yes	107 (31.2)	616 (23.9)	8.590	0.003
No	236 (68.8)	1959 (76.1)		
Exertion in defecation ^†^	Yes	Never		
Yes	203 (59.2)	1334 (51.8)	6.645	0.010
No	140 (40.8)	1242 (48.2)		
Sensation of incomplete emptying ^†^	Yes	Never		
Yes	246 (71.7)	1740 (67.6)	2.366	0.124
No	97 (28.3)	834 (32.4)		
Sensation of obstruction in defecation ^†^	Yes	Never		
Yes	193 (56.3)	1221 (47.5)	9.413	0.002
No	150 (43.7)	1352 (52.5)		
Manipulation ^†^	Yes	Never		
Yes	46 (13.3)	291 (11.2)	1.301	0.254
No	299 (86.7)	2296 (88.8)		
Diarrhea ^†^	Yes	Never		
Yes	190 (55.6)	1446 (56.4)	0.096	0.757
No	152 (44.4)	1116 (43.6)		

FBDs: functional bowel diseases. ^†^: Pearson’s χ^2^ test. *p* < 0.05 was considered to be of significance.

**Table 5:** The association between passive smoking and frequency of symptoms among FBD patients (N = 236).

Symptoms	Passive Smoking (%)		χ^2^	*p* Value
Abdominal pain ^†^	Yes	Never		
High frequency	8 (25.8)	44 (31.0)	0.325	0.569
Low frequency	23 (74.2)	98 (69.0)		
Abdominal bloating and/or distention ^†^	Yes	Never		
High frequency	7 (29.2)	40 (48.8)	2.894	0.089
Low frequency	17 (70.8)	42 (51.2)		
Hard stool ^†^	Yes	Never		
High frequency	24 (60.0)	57 (39.6)	5.295	0.021
Low frequency	16 (40.0)	87 (60.4)		
Decreased defecation ^†^	Yes	Never		
High frequency	11 (47.8)	29 (37.7)	0.762	0.383
Low frequency	12 (52.2)	48 (62.3)		
Exertion in defecation ^†^	Yes	Never		
High frequency	20 (48.8)	38 (28.8)	5.611	0.018
Low frequency	21 (51.2)	94 (71.2)		
Sensation of incomplete emptying ^†^	Yes	Never		
High frequency	21 (45.7)	66 (41.3)	0.284	0.594
Low frequency	25 (54.3)	94 (58.8)		
Sensation of obstruction in defecation ^†^	Yes	Never		
High frequency	22 (51.2)	44 (33.8)	4.106	0.043
Low frequency	21 (48.8)	86 (66.2)		
Manipulation ^§^	Yes	Never		
High frequency	6 (66.7)	24 (43.6)	-	0.285
Low frequency	3 (33.3)	31 (56.4)		
Diarrhea ^†^	Yes	Never		
High frequency	19 (55.9)	67 (46.9)	0.896	0.344
Low frequency	15 (44.1)	76 (53.1)		

FBDs: functional bowel diseases. ^†^: Pearson’s χ^2^ test; ^§^: Fisher’s exact test. *p* < 0.05 was considered to be of significance.

## Data Availability

The raw data supporting the conclusions of this article will be made available by the authors upon request.

## References

[1] Ma C., Congly S.E., Novak K.L., Belletrutti P.J., Raman M., Woo M., Andrews C.N., Nasser Y. (2021). Epidemiologic Burden and Treatment of Chronic Symptomatic Functional Bowel Disorders in the United States: A Nationwide Analysis. Gastroenterology.

[2] Sperber A.D., Bangdiwala S.I., Drossman D.A., Ghoshal U.C., Simren M., Tack J., Whitehead W.E., Dumitrascu D.L., Fang X., Fukudo S. (2021). Worldwide Prevalence and Burden of Functional Gastrointestinal Disorders, Results of Rome Foundation Global Study. Gastroenterology.

[3] Gwee K.A., Ghoshal U.C., Chen M. (2018). Irritable bowel syndrome in Asia: Pathogenesis, natural history, epidemiology, and management. J. Gastroenterol. Hepatol..

[4] Chen Z., Peng Y., Shi Q., Chen Y., Cao L., Jia J., Liu C., Zhang J. (2022). Prevalence and Risk Factors of Functional Constipation According to the Rome Criteria in China: A Systematic Review and Meta-Analysis. Front. Med..

[5] Jiang C.X., Li Z.Z., Chen P., Chen L.Z. (2015). Prevalence of Depression Among College-Goers in Mainland China: A Methodical Evaluation and Meta-Analysis. Medicine.

[6] Ranasinghe N., Devanarayana N.M., Benninga M.A., van Dijk M., Rajindrajith S. (2017). Psychological maladjustment and quality of life in adolescents with constipation. Arch. Dis. Child..

[7] Stok F.M., Renner B., Clarys P., Lien N., Lakerveld J., Deliens T. (2018). Understanding Eating Behavior during the Transition from Adolescence to Young Adulthood: A Literature Review and Perspective on Future Research Directions. Nutrients.

[8] Hickie I.B., Scott E.M., Cross S.P., Iorfino F., Davenport T.A., Guastella A.J., Naismith S.L., Carpenter J.S., Rohleder C., Crouse J.J. (2019). Right care, first time: A highly personalised and measurement-based care model to manage youth mental health. Med. J. Aust..

[9] Neumark-Sztainer D., Wall M.M., Chen C., Larson N.I., Christoph M.J., Sherwood N.E. (2018). Eating, Activity, and Weight-related Problems from Adolescence to Adulthood. Am. J. Prev. Med..

[10] Suldo S.M., Shaunessy E., Thalji A., Michalowski J., Shaffer E. (2009). Sources of stress for students in high school college preparatory and general education programs: Group differences and associations with adjustment. Adolescence.

[11] Zeng J., Yang S., Wu L., Wang J., Wang Y., Liu M., Zhang D., Jiang B., He Y. (2016). Prevalence of passive smoking in the community population aged 15 years and older in China: A systematic review and meta-analysis. BMJ Open.

[12] Zia J.K., Lenhart A., Yang P.L., Heitkemper M.M., Baker J., Keefer L., Saps M., Cuff C., Hungria G., Videlock E.J. (2022). Risk Factors for Abdominal Pain-Related Disorders of Gut-Brain Interaction in Adults and Children: A Systematic Review. Gastroenterology.

[13] Baspinar M.M., Basat O. (2022). Frequency and severity of irritable bowel syndrome in cigarette smokers, Turkey 2019. Tob. Induc. Dis..

[14] Nishikawa A., Tanaka K., Miyake Y., Nagata C., Furukawa S., Andoh A., Yokoyama T., Yoshimura N., Mori K., Ninomiya T. (2022). Active and passive smoking and risk of ulcerative colitis: A case-control study in Japan. J. Gastroenterol. Hepatol..

[15] Drossman D.A. (2016). Functional Gastrointestinal Disorders: History, Pathophysiology, Clinical Features and Rome IV. Gastroenterology.

[16] Palsson O.S., Whitehead W., Tornblom H., Sperber A.D., Simren M. (2020). Prevalence of Rome IV Functional Bowel Disorders Among Adults in the United States, Canada, and the United Kingdom. Gastroenterology.

[17] Liu M., Guo W., Cai Y., Yang H., Li W., Yang L., Lai X., Fang Q., Ma L., Zhu R. (2020). Personal exposure to fine particulate matter and renal function in children: A panel study. Environ. Pollut..

[18] Gadzella B.M. (1994). Student-Life Stress Inventory: Identification of and reactions to stressors. Psychol. Rep..

[19] Jones D.T., Osterman M.T., Bewtra M., Lewis J.D. (2008). Passive smoking and inflammatory bowel disease: A meta-analysis. Am. J. Gastroenterol..

[20] Poirier A.E., Ruan Y., Grevers X., Walter S.D., Villeneuve P.J., Friedenreich C.M., Brenner D.R., ComPARe Study Team (2019). Estimates of the current and future burden of cancer attributable to active and passive tobacco smoking in Canada. Prev. Med..

[21] Jordan R.E., Cheng K.K., Miller M.R., Adab P. (2011). Passive smoking and chronic obstructive pulmonary disease: Cross-sectional analysis of data from the Health Survey for England. BMJ Open.

[22] Li J., Du Y.J., Wang H.L., Du J.Y., Qu P.F., Zhang R., Guo L.Q., Yan H., Dang S.N. (2020). Association between maternal passive smoking during perinatal period and congenital heart disease in their offspring-based on a case-control study. Zhonghua Liu Xing Bing Xue Za Zhi.

[23] Duan L., Wu A.H., Sullivan-Halley J., Bernstein L. (2009). Passive smoking and risk of oesophageal and gastric adenocarcinomas. Br. J. Cancer.

[24] Talley N.J., Powell N., Walker M.M., Jones M.P., Ronkainen J., Forsberg A., Kjellström L., Hellström P.M., Aro P., Wallner B. (2021). Role of smoking in functional dyspepsia and irritable bowel syndrome: Three random population-based studies. Aliment. Pharmacol. Ther..

[25] Miglis M.G. (2016). Autonomic dysfunction in primary sleep disorders. Sleep Med..

[26] Schey R., Dickman R., Parthasarathy S., Quan S.F., Wendel C., Merchant J., Powers J., Han B., van Handel D., Fass R. (2007). Sleep deprivation is hyperalgesic in patients with gastroesophageal reflux disease. Gastroenterology.

[27] Zhou E., Ma S., Kang L., Zhang N., Wang P., Wang W., Nie Z., Chen M., Xu J., Sun S. (2023). Psychosocial factors associated with anxious depression. J. Affect. Disord..

[28] Freese J., Klement R.J., Ruiz-Nunez B., Schwarz S., Lotzerich H. (2017). The sedentary (r)evolution: Have we lost our metabolic flexibility?. F1000Research.

[29] Klement R.J., Pazienza V. (2019). Impact of Different Types of Diet on Gut Microbiota Profiles and Cancer Prevention and Treatment. Medicina.

